# Reconstruction of Defects After Fournier Gangrene: A Systematic Review

**Published:** 2015-05-26

**Authors:** Laurel S. Karian, Stella Y. Chung, Edward S. Lee

**Affiliations:** Division of Plastic Surgery, Rutgers–New Jersey Medical School, Newark

**Keywords:** Fournier gangrene, systematic review, scrotal and perineal defects, scrotal advancement flap, skin graft

## Abstract

**Background:** Reconstruction of scrotal defects after Fournier gangrene is often achieved with skin grafts or flaps, but there is no general consensus on the best method of reconstruction or how to approach the exposed testicle. We systematically reviewed the literature addressing methods of reconstruction of Fournier defects after debridement. **Methods:** PubMed and Cochrane databases were searched from 1950 to 2013. Inclusion criteria were reconstruction for Fournier defects, patients 18 to 90 years old, and reconstructive complication rates reported as whole numbers or percentages. Exclusion criteria were studies focused on methods of debridement or other phases of care rather than reconstruction, studies with fewer than 5 male patients with Fournier defects, literature reviews, and articles not in English. **Results:** The initial search yielded 982 studies, which was refined to 16 studies with a total pool of 425 patients. There were 25 (5.9%) patients with defects that healed by secondary intention, 44 (10.4%) with delayed primary closure, 36 (8.5%) with implantation of the testicle in a medial thigh pocket, 6 (1.4%) with loose wound approximation, 96 (22.6%) with skin grafts, 68 (16.0%) with scrotal advancement flaps, 128 (30.1%) with flaps, and 22 (5.2%) with flaps or skin grafts in combination with tissue adhesives. Four outcomes were evaluated: number of patients, defect size, method of reconstruction, and wound-healing complications. **Conclusions:** Most reconstructive techniques provide reliable coverage and protection of testicular function with an acceptable cosmetic result. There is no conclusive evidence to support flap coverage of exposed testes rather than skin graft. A reconstructive algorithm is proposed. Skin grafting or flap reconstruction is recommended for defects larger than 50% of the scrotum or extending beyond the scrotum, whereas scrotal advancement flap reconstruction or healing by secondary intention is best for defects confined to less than 50% of the scrotum that cannot be closed primarily without tension.

Fournier gangrene is a necrotizing fasciitis of the perineum and external genitalia that rapidly spreads along contiguous fascia. In addition to cardiopulmonary support and broad-spectrum antibiotics, the standard treatment involves aggressive debridement of necrotic tissue.^1^ Multiple debridements may be required, often resulting in significant soft-tissue loss requiring reconstruction. The goals of reconstructing Fournier defects are to provide protective coverage of the testes, preserve testicular function, and gain acceptable cosmetic results with minimal associated morbidity and mortality. Fournier gangrene often affects patients with significant comorbidities including diabetes, alcoholism, and advanced age^2^; thus, a technically simple procedure is preferred. There is no general consensus on the best method of reconstruction or how to approach the exposed testicle. We systematically reviewed the literature addressing methods of reconstruction for Fournier defects after final debridement.

## METHODS

### Search strategy

PubMed and Cochrane databases were thoroughly searched by the authors (January 1, 1950, to September 20, 2013). In addition, bibliographies of each relevant citation were reviewed for additional sources. The PubMed search was conducted using 2 groups of subject headings and key words concatenated together to connect disease and reconstruction, as enumerated in the following:

1. Subject headings related to disease “Fournier gangrene/surgery and scrotum/surgery” and key words “Fournier's” and “scrotum”; and
2. Subject headings related to reconstruction: “surgical flaps,” “perforator flap,” “free tissue flaps,” “reconstructive surgical procedures,” “surgery, plastic,” “wound closure techniques,” “grafting, tissue,” and key words “flap,” “reconstruction,” “graft,” “ALT,” “anterolateral,” “gracilis,” “medial thigh,” “advancement,” “pudendal.”

**Figure 1:**
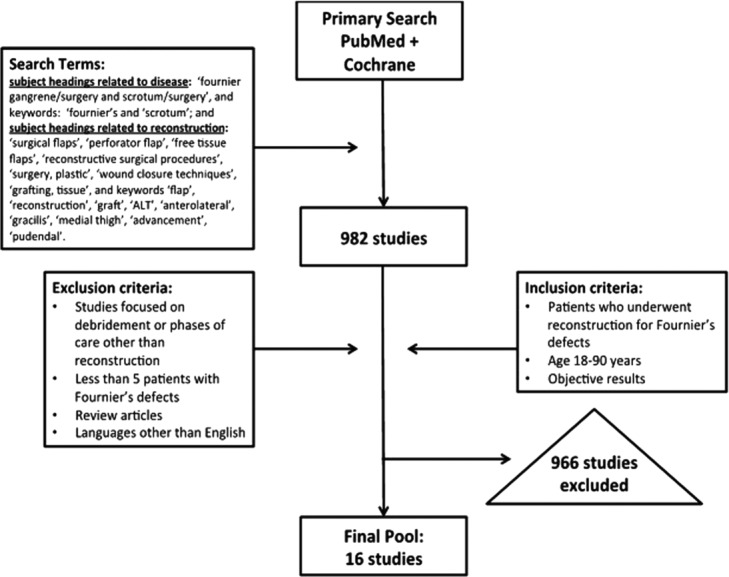
Citation flow diagram for the review process.

The initial PubMed search yielded 982 studies that included at least 1 search term from each group. The Cochrane database did not yield any studies, which was expected, given that there are no prospective studies on this subject. Two authors independently reviewed all the abstracts of these 982 studies and subsequently included or excluded studies based on the inclusion and exclusion criteria. Articles of abstracts that met criteria were reviewed as a second stage. Discrepancies between the authors were discussed, and a third senior author made the decision as to whether the study should be included or excluded. After revising the list based on our criteria, 16 studies that were published between 1984 and 2013 were selected. The search algorithm is shown in Figure 1.

### Inclusion/exclusion criteria

The authors included studies that were published in scientific journals and provided information about patients who had undergone reconstruction for Fournier defects and studies that addressed objective results in patients who were older than 18 years and younger than 90 years. The authors excluded studies that were focused on methods of debridement or other phases of care rather than reconstruction, studies focused on negative pressure wound therapy, studies with fewer than 5 patients with Fournier defects, literature reviews, and articles that were published in languages other than English. Studies with female patients or patients with defects resulting from disease processes other than Fournier gangrene were included as long as the number of male patients with Fournier defects was greater than 5 and represented the majority of the patient pool.

## RESULTS

The electronic literature search yielded 982 studies, 966 of which were excluded on the basis of our criteria. The final pool comprised 16 studies with a total of 425 patients (Table 1). Of these patients, there were 25 (5.9%) with defects that healed by secondary intention, 44 (10.4%) with delayed primary closure, 36 (8.5%) with implantation of the testicle in a medial thigh pocket, 6 (1.4%) with loose wound approximation, 96 (22.6%) with skin grafts, 68 (16.0%) with scrotal advancement flaps, 128 (30.1%) with flaps, and 22 (5.2%) with flaps or grafts in combination with tissue adhesives (Table 2).

Four outcomes were evaluated on the basis of data extracted from the 16 included studies: number of patients, defect size, method of reconstruction, and wound-healing complications. Because of the differences in reported outcomes among the included studies, outcomes could not be combined as a meta-analysis.

### Healing by secondary intention

Two studies included in this systematic review evaluated a total of 25 (5.9%) patients with defects that healed by secondary intention.^3^^,^^4^ The indication for healing by secondary intention in both studies was a small defect confined to the scrotum. One study compared healing by secondary intention with loose U-stitch approximation for defects confined to less than 50% of the scrotum and found that patients with defects that healed by secondary intention required longer hospital stays than patients treated with loose wound closure, although the result was not significant (mean = 28.9 days vs 11.0 days; *P* = .385).^4^

### Loose wound approximation

Akilov et al^4^ performed loose approximation of the scrotum for 6 (1.4%) defects confined to less than 50% of the scrotum and compared this method with healing by secondary intention for 14 defects. Loose wound approximation was performed at the time of debridement with a nonabsorbable monofilament suture by U-stitch approximation of the wound edges, and a Penrose drain was placed to permit drainage and prevent premature wound contraction. Additional wet-to-dry dressing changes and irrigation were performed postoperatively. A shorter hospital stay was observed for patients who underwent loose wound approximation than for those with healing by secondary intention, although the result was not significant. Of note, 3 patients in this study required orchiectomy due to late epidimoorchitis.^4^

### Subcutaneous thigh pockets

Three of the selected studies evaluated a total of 36 (8.5%) patients with defects that were managed by permanent or temporary placement of testes in medial thigh pockets.^5^^-^7 Bhatnager et al^5^ described poor cosmesis and low patient satisfaction after medial thigh pockets in 26 patients. Badejo^6^ reported normal sperm counts and normal histology before and after thigh implantation in 10 patients, although 2 patients lost 1 testis each due to spermatic cord necrosis.

### Local scrotal advancement flap

Four studies included in this systematic review described successful reconstruction of scrotal defects with local scrotal advancement flaps, for a total pool of 68 patients.^3^^,^^8^^-^10 Chen et al^8^ described success using the scrotal advancement flap for 11 patients with scrotal defects smaller than half the surface area of the scrotum. Complications included partial flap loss in 1 patient and wound edge necrosis in 2 patients. The authors advised caution with use of this flap for defects larger than half the scrotum because the closure should be tension free.^8^ Ferreira et al^9^ included muscle in the advancement flap, with 10 patients undergoing scrotal musculocutaneous flaps for small scrotal defects.

While most authors recommend this flap for reconstruction of small scrotal defects, Parkash and Gajendran^10^ reported good outcomes in 40 patients with defects confined to the scrotum that were treated with mobilization of scrotal skin to cover the testes. Even when the majority of the scrotum was involved, they performed mobilization of the surrounding scrotal rim with the addition of skin grafting alone when the defect extended beyond the scrotum in 3 patients. Four patients in this group experienced wound dehiscence that healed spontaneously.^10^

### Split-thickness skin grafting

Nine studies in this systematic review evaluated a total of 96 (22.6%) patients who underwent skin grafting for Fournier defects.^3^^-^11 Five of these studies reported quantitative results of wound-healing complications. Ferreira et al^9^ cited good-quality take in all skin grafts, used alone or in combination with flaps, in 5 small perineal defects and 7 penile defects. Carvalho et al^3^ described infectious complications in 3 of 16 (18%) skin grafts. Chen et al^8^ cited partial graft loss in 1 of 9 (11%) skin grafts. Tan et al^7^ reported 100% take in 23 of 24 skin grafts, with 1 patient who experienced infection and scarring. The authors also reported noticeable adhesions in 5 (18%) patients. However, they noted that with the use of skin emollients and massage, the testes gradually softened and were able to move independently at 6 months.^7^ Akilov et al^4^ reported ipsilateral orchiectomy in 1 patient who developed chronic pain after scrotal skin graft reconstruction.

### Flaps

A total of 11 articles evaluated flap reconstruction for Fournier defects, with a pool of 128 (30.1%) patients.^3^^,^^5^^,^^7^^-^9^,^^11^^-^16 Variations of medial thigh fasciocutaneous flaps are described in 3 articles (39 patients). Ferreira et al^9^ reported partial dehiscence requiring reoperation in 5 of 26 patients who underwent reconstruction with a superomedial thigh fasciocutaneous flap. Bhatnager et al^5^ reported wound-healing complications in 2 of 10 patients who underwent reconstruction with a fasciocutaneous rotation flap from the anteromedial upper thigh (flap necrosis and infection with subsequent rejection). Tan et al^7^ performed bilateral medial thigh flaps in 1 patient and found the result to be a cosmetic improvement compared with testicular thigh pouches but with a scrotal sac that was poorly formed.

**Figure 2:**
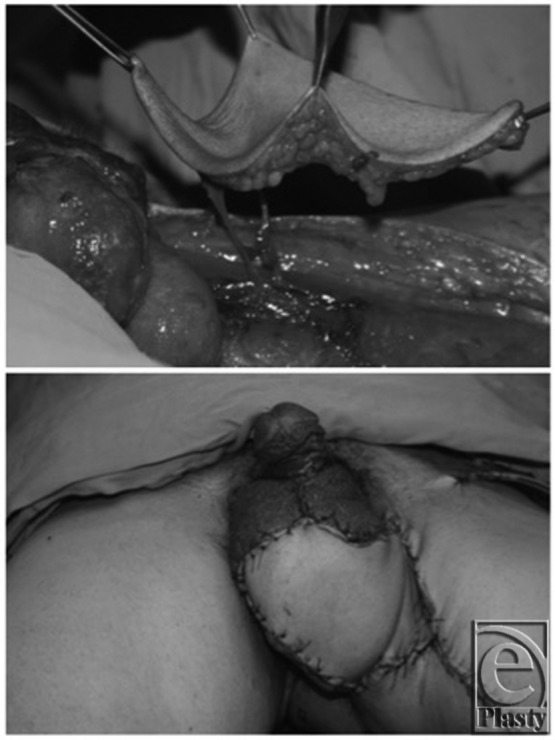
Medial circumflex femoral artery perforator flap (top). Early postoperative view (bottom). Reprinted with permission from Coskunfirat et al.^12^

Coskunfirat et al^12^ performed a medial circumflex femoral artery perforator flap in 7 patients. They reported ease of flap transfer to the scrotal area, ability to thin the skin of the flap for improved scrotal contour, and low donor-site morbidity due to preservation of the muscle (Fig 2). They found good flap survival in all cases. Complications included wound dehiscence in 2 patients, and poor sensation in all cases.^12^

**Figure 3:**
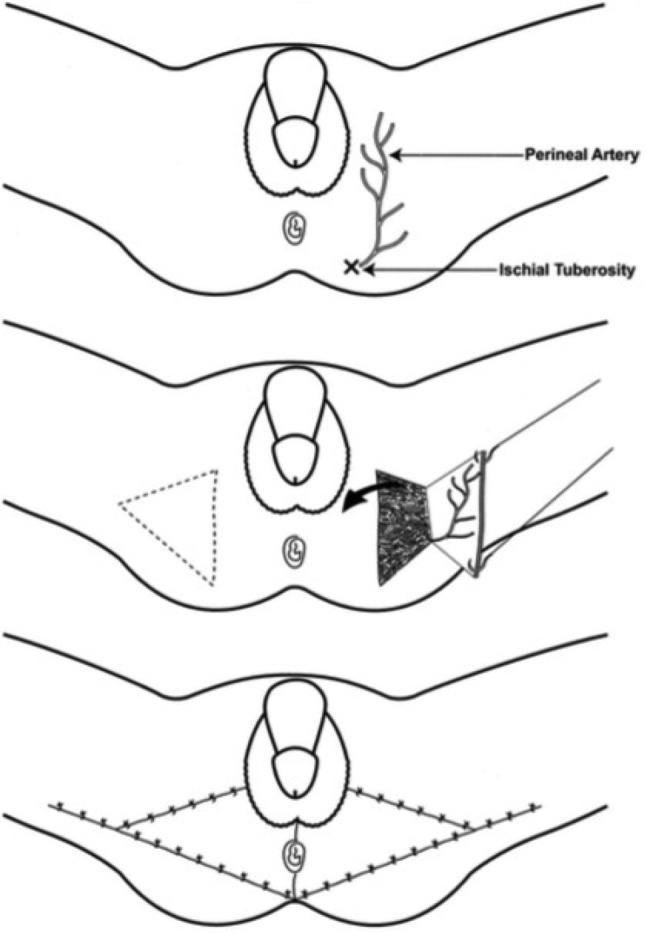
Anatomic illustration of the pudendal thigh flap with a V-Y design. (a) The flap pedicle. (b) Flap design and elevation. (c) The flap inset. Reprinted with permission from El-Khatib.^11^

Pudendal thigh flaps are described in 3 of the articles included in this systematic review with a total pool of 21 patients. El-Khatib^11^ reported excellent flap survival and intact sensation after reconstruction with a V-Y island fasciocutaneous pudendal thigh flap in 8 patients. The flap was elevated bilaterally on the terminal branches of the superficial perineal artery with inclusion of the superficial perineal nerve (Fig 3).^11^ Karaçal et al^13^ described a neurovascular pedicled pudendal thigh flap in 8 patients, also supplied by the superficial perineal artery with preservation of the superficial perineal nerve in the flap. Instead of a V-Y design, the authors used a peninsular transposition design. Chen et al^8^ reported a good cosmetic outcome after reconstruction with a subcutaneously tunneled pudendal thigh flap in 5 patients.

Gracilis flap reconstruction was reported in 3 studies with a total pool of 17 patients. Chen et al^8^ used a gracilis flap for 3 patients with a dead cavity in the perineal area. Hsu et al^14^ reported complications in only 2 of 17 patients (hematoma and infection) after reconstruction with a gracilis V-Y myofasciocutaneous advancement flap with wide incorporation of the perigracilis fascia in 8 patients. The authors stated that the flap has improved vascular supply to the cutaneous portion of the flap due to incorporation of septocutaneous perforators traveling around the gracilis muscle, which is well described in the literature.^17^^-^19 Lee et al^15^ described the combination of a unilateral gracilis muscle flap with an internal pudendal artery perforator flap in 6 patients, with the addition of a skin graft when both testicles were involved. They reported no wound dehiscence, lymphedema, or venous congestion and reported satisfaction with the functional and aesthetic outcome in all cases.^15^

Other described flaps include the pedicled anterolateral thigh (ALT) and vertical rectus abdominis myocutaneous (VRAM) flaps. Two studies described reconstruction with a pedicled ALT flap with a total pool of 12 patients. Chen et al^8^ reported good flap survival and an aesthetically pleasing outcome with the use of a pedicled ALT flap transposed subcutaneously to cover 3 defects involving the scrotum and the perineum. Spyropoulou et al^16^ used a pedicled ALT flap in 9 patients with perinoscrotal defects. The authors stated that although the flap appeared bulky in the initial postoperative period, the edema subsided in the long term, leaving a more natural looking scrotum.^16^ In addition, Tan et al^7^ reported good coverage with a VRAM flap but stated that it did not mimic the normal scrotum in appearance. Carvalho et al^3^ reported complications in 4 of 21 skin flaps, with 2 cases of flap infection and 2 cases of flap loss.

### Tissue adhesives

Two studies evaluated the use of tissue adhesives in combination with 22 (5.2%) skin grafts or flaps for reconstruction of Fournier defects, with a total of 18 skin grafts and 4 flaps.^20^^,^^21^ Both authors reported excellent results with 100% skin graft take and no incidences of infection, hematoma, or seroma. One of the flaps developed breakdown at 25% of the wound edges.

### Aesthetics

Few of the studies presented in this systematic review reported aesthetic outcomes of reconstruction. Twelve studies including 233 cases provided some assessment regarding aesthetic outcomes, although these assessments were largely subjective. All 26 thigh pouches and 4 orchiectomies were determined to be aesthetically unsatisfactory due to low ratings in patient satisfaction.^5^^,^^7^ Aesthetic outcomes were evaluated after skin grafting in 5 studies, with a total of 82 successful cases.^3^^,^^5^^,^^7^^,^^8^^,^^21^ All authors reported satisfactory results, with few reports of unacceptable scar contracture. Good cosmesis was also reported after reconstruction with scrotal advancement flaps, with good skin color match and natural scrotal contour in 9 of 12 cases.^8^ Flap reconstruction is generally thought to be less cosmetically acceptable than skin grafting, mainly due to excessive bulkiness. However, acceptable cosmesis has been reported in all cases of flap reconstruction included in this systematic review.^3^^,^^5^^,^^7^^-^9^,^^11^^-^16 Coskunfirat et al^12^ stated that the medial circumflex femoral artery perforator flap can be thinned, resulting in improvement in scrotal contour compared with other fasciocutaneous flaps, although they admitted that this flap was still a poor color match. Lee et al^15^ reported adequate bulkiness and excellent scrotal contour after 6 gracilis flaps combined with internal pudendal artery perforator fasciocutaneous flaps. Hair-bearing skin may be used to improve the cosmetic result of flap reconstruction.^7^

### Testicular function

Only 4 studies in this systematic review provided assessment of testicular function outcomes after scrotal reconstruction, the majority of which were subjective. Three studies reported overall patient satisfaction with the functional outcome after scrotal reconstruction with flap reconstruction but did not explain how this was assessed.^11^^,^^14^^,^^15^ Only 1 study reported objective outcomes based on testicular biopsy before and after implantation of the testes in the medial thigh, with normal sperm counts and normal histology reported before and 6 months after implantation in the thigh.^6^

## DISCUSSION

The “replace like with like” principle is important for tissue functionality as well as aesthetic purposes. In Fournier gangrene, reconstruction of scrotal, penile, and perineal defects after surgical debridement is a challenge. These organs have unique texture, color, and contour that are difficult to recreate. The best cosmetic and functional results are achieved by primary closure without tension, which is only possible for very small defects of the scrotum. Any tension on the closure is an indication for reconstruction.

Fournier gangrene tends to affect older patients with significant medical comorbidities such as diabetes and alcoholism, which makes these patients at higher risk for surgical procedures, especially under general anesthesia. Reconstruction should ideally be limited to 1 stage and should be technically simple and low cost. Orchiectomy should never be required, because the testicles and the spermatic cord have blood supply and venous drainage distinct from the skin and fascia of the perineum and the scrotum.

Fournier defects may be allowed to heal by secondary intention if they are relatively small and confined to less than 50% of the scrotum.^3^^,^^4^^,^^22^^,^^23^ We recommend this method for small scrotal defects, as long as the surgeon is aware of the potential for prolonged healing time, contracture, and deformity. We do not recommend loose wound approximation at the time of debridement because of the associated risk of worsening infection.

Implantation of testes in subcutaneous thigh pockets has historically been used either for permanent coverage of testes or as a first-stage procedure to protect the testes until final reconstruction is performed. Although this method is simple and carries low donor-site morbidity, it is considered to be cosmetically and functionally unacceptable due to concerns over temperature regulation, psychological effects, and potential for pain.^5^^-^7 The appearance of testes hidden in the thigh is completely unnatural, especially for younger patients. In addition, fertility may be compromised due to high temperatures in the thigh.^24^^,^^25^ Objective evidence regarding testicular histology and spermatogenesis is limited but suggests that placement of testes in the thigh is detrimental to both hormonal production by Leydig cells and spermatogenesis.^26^^,^^27^

**Figure 4:**
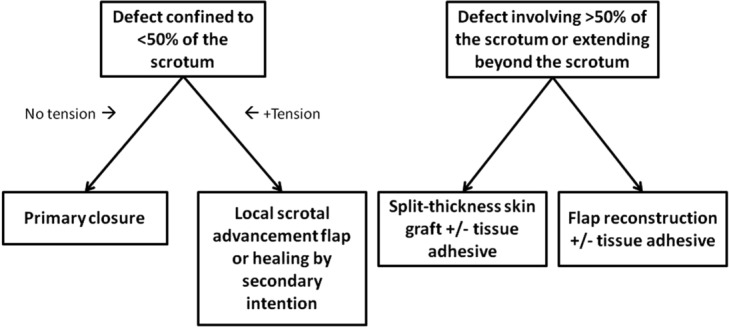
Proposed algorithm for reconstruction of Fournier defects.

It is known that as little as one-third of residual scrotum can be expanded to resurface the entire scrotum.^28^ This method involves undermining in all directions around the scrotal defect in the subcutaneous plane. Some authors advocate elevation as a musculocutaneous flap, with incorporation of the dartos muscle by dissecting between the dartos muscle and external spermatic fascia.^28^ We recommend local scrotal advancement flaps, with or without incorporation of the dartos muscle, for patients with defects confined to less than 50% of the scrotum that cannot be closed primarily without tension (Fig 4). The flap is technically simple with low donor-site morbidity and carries a low overall complication rate.^3^^,^^8^^-^10

**Figure 5:**
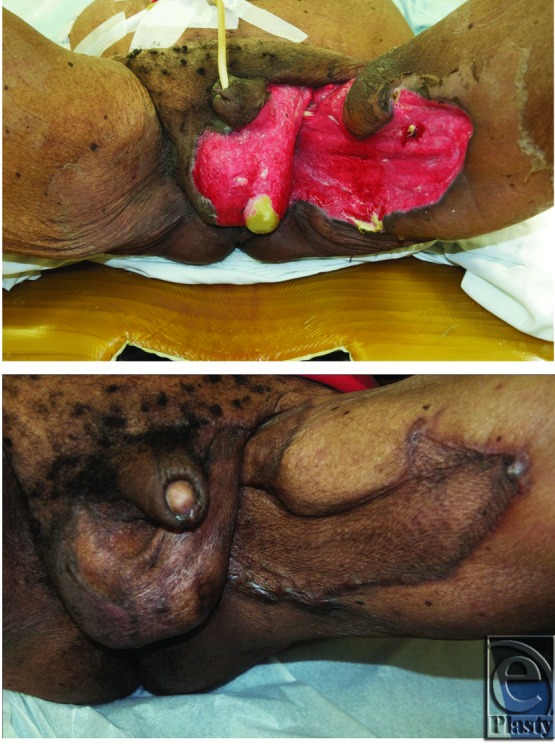
Preoperative (top) and postoperative (bottom) split-thickness skin grafting after Fournier gangrene.

For defects larger than 50% of the scrotum or extending beyond the scrotum, we recommend split-thickness skin grafting or flap reconstruction (Fig 4). Skin grafting is a simple and technically easy procedure, can be performed in a single stage, and can cover large defects with acceptable functional and cosmetic results.^3^^,^^7^^,^^29^ The thin skin resembles normal scrotal skin and keeps the testes cool, preventing testicular dysfunction. The color and shape are close to normal scrotal skin. A healthy bed of granulation tissue is a prerequisite. Usually, the wound bed is prepared for grafting by tangential excision of redundant granulation tissue, pulse lavage, or other method of wound bed preparation. The testicles are sutured together with interrupted absorbable sutures, and a 2:1 meshed split-thickness skin graft is applied and stapled or sutured in place. The graft can be bolstered in place to prevent shear (Fig 5).

The most common complications of skin grafting are contraction and graft loss due to bleeding, shearing, or infection. In addition, some authors feel that the thin grafted skin is potentially vulnerable to trauma and may not provide as much protection of the testes compared with flap reconstruction.^5^ Pain or discomfort due to lack of mobility between grafted skin and testes has also been reported.^29^^,^^30^ Finally, some authors state that skin grafting cannot be performed if the testes have been stripped of the tunica vaginalis.^24^ However, we have not found this to be the case in our experience. We find these reported complications to be acceptable, given the simplicity and low donor-site morbidity.

Reported benefits of flap reconstruction include durable protection of the testes, provision of immediate coverage without waiting for granulation tissue formation, and lower incidence of contracture. Opinions about cosmesis after flap versus skin graft are conflicting. Some authors feel that the cosmetic results of flap reconstruction are suboptimal compared with skin grafting because flaps are much thicker than scrotal skin,^7^ whereas others support that acceptable cosmetic results are attained.^3^^,^^5^,^7^^-^9,11^-^16 We recognize that flap reconstruction may be chosen as an option for Fournier defects larger than 50% of the scrotum or extending beyond the scrotum. However, the surgeon must be wary that flap reconstructive procedures are longer and more complex than skin grafts and may be associated with increased donor-site and recipient-site morbidity. Complications include partial or total flap loss, wound dehiscence and donor-site scarring, seroma, and hematoma. In addition, testicular function may be compromised because of exposure to higher temperatures after flap reconstruction.

Use of tissue adhesives in securing skin grafts or flaps in reconstruction of Fournier defects is thought to be helpful in grafting over complex contours and in decreasing incidence of hematoma and seroma. In addition, sealants decrease the need for staples or sutures, which may be beneficial in contaminated wounds and obviate the need for removal under anesthesia. We find no disadvantage to the use of fibrin sealant or cyanoacrylate glue to aid in securing skin grafts or flaps, although larger studies are warranted to show that these adhesives significantly decrease complication rates.

There are several limitations to our study that should be addressed. First, we were not able to find any prospective randomized controlled studies that met our inclusion criteria. All of the studies included are retrospective, which we realize restricts the strength of our conclusions. We chose to include studies with female patients as long as the male patients represent the majority of the patient pool. There were only 2 female patients included in our final patient pool, from 2 different articles. We feel that of our total pool of 425 patients, the addition of these articles was beneficial to our discussion and analysis.

There is substantial room for further research regarding outcomes of Fournier reconstruction. There is little report on patient-reported outcomes within the articles in this systematic review. Assessment of patient-reported outcomes with regard to patient satisfaction in terms of cosmesis, function, and mobility would be useful in future research.

## CONCLUSION

Although various methods of scrotal reconstruction after Fournier gangrene have been described, reliable coverage and protection of testicular function with an acceptable cosmetic result are seen with most reconstructive techniques. Data from the individual studies are difficult to interpret because of the variability in reported complications. We conclude that the simplest and least costly procedure is ideal for reconstructing these defects, especially since Fournier gangrene occurs predominantly in patients with significant comorbidities who may be at an increased operative risk in longer or multiple procedures. There is no conclusive evidence to support flap coverage of exposed testes rather than skin graft. An algorithm is proposed for reconstruction based on the complication rates and observations reported by the authors cited in this systematic review. Skin grafting or flap reconstruction is recommended for defects larger than 50% of the scrotum or extending beyond the scrotum, whereas scrotal advancement flap reconstruction or healing by secondary intention is best for defects confined to less than 50% of the scrotum that cannot be closed primarily without tension. Further assessment of patient satisfaction regarding testicular function, cosmesis, and mobility is warranted.

## Figures and Tables

**Table 1:** Characteristics of the included studies*

Author	Number of cases	Reconstructive methods	Complication rate, % (*n*)	Study design	Level of evidence
Parkash and Gajendran^10^	43	Scrotal advancement flap (*n* = 40); skin graft (*n* = 3)	9 (4)	Retrospective study	4
Badejo^6^	16	Skin graft (*n* = 6); medial thigh pocket (*n* = 10)	N/A	Retrospective study	4
El-Khatib^11^	11	Pudendal thigh flap (*n* = 8); skin graft (*n* = 3)	N/A	Retrospective study	4
Morris et al^20^	7	Skin graft + fibrin sealant (*n* = 3); flap + fibrin sealant (*n* = 4)	14 (1)	Retrospective study	4
Ferreira et al^9^	40	Superomedial thigh flap (*n* = 26); scrotal musculocutaneous flap (*n* = 10); skin graft (*n* = 4)	Superomedial thigh flaps: 19% (5); skin grafts: N/A	Retrospective study	4
Karaçal et al^13^	8	Pudendal thigh flap ± skin graft	N/A	Retrospective study	4
Hsu et al^14^	8	Gracilis flap	25 (2)	Retrospective study	4
Carvalho et al^3^	67	Healing by secondary intention (*n* = 11), local scrotal advancement (*n* = 16), skin graft (*n* = 19), flap (*n* = 21)	10 (7)	Retrospective study	4
Bhatnager et al^5^	102	Delayed primary closure (*n* = 44); medial thigh pocket (*n* = 26); skin graft (*n* = 20); fasciocutaneous thigh flap (*n* = 12)	2 (2)	Retrospective study	4
Chen et al^8^	31	Scrotal advancement flap (*n* = 11); skin graft (*n* = 9); pudendal thigh flap (*n* = 5); gracilis flap (*n* = 3); ALT flap (*n* = 3)	16 (5)	Retrospective study	4
Tan et al^7^	27	Skin graft (*n* = 24); VRAM flap (*n* = 1); medial thigh flap (*n* = 1); medial thigh pocket (*n* = 1)	4 (1)	Retrospective study	4
Coskunfirat et al^12^	7	Medial circumflex femoral artery perforator flap	28 (2)	Retrospective study	4
Lee et al^15^	6	Gracilis flap + internal pudendal artery perforator flap	N/A	Retrospective study	4
Sivrioğlu et al^21^	15	Skin graft + cyanoacrylate glue	0 (0)	Retrospective study	4
Akilov et al^4^	28	Loose wound approximation (*n* = 6); healing by secondary intention (*n* = 14); skin graft (*n* = 8)	14 (4)	Retrospective study	4
Spyropoulou et al^16^	9	ALT flap ± skin graft	22 (9)	Retrospective study	4

*ALT indicates anterolateral thigh; N/A, not applicable; and VRAM, vertical rectus abdominis myocutaneous.

**Table 2:** Methods of wound closure in the included studies*

Method of wound closure	Studies	Total pooled number of patients treated
Healing by secondary intention	Carvalho et al,^3^ Akilov et al^4^	25
Delayed primary closure	Bhatnager et al^5^	40
Orchidectomy + delayed closure	Bhatnager et al^5^	4
Medial thigh pocket implantation	Badejo,^6^ Bhatnager et al,^5^ Tan et al^7^	36
Loose wound approximation	Akilov et al^4^	6
Skin graft	Parkash and Gajendran,^10^ Badejo,^6^ El-Khatib,^11^ Ferreira et al,^9^ Carvalho et al,^3^ Bhatnager et al,^5^ Chen et al,^8^ Tan et al,^7^ Akilov et al^4^	96
Scrotal advancement flap	Parkash and Gajendran,^10^ Carvalho et al,^3^ Ferreira et al,^9^ Chen et al^8^	78
Flap	El-Khatib,^11^ Carvalho et al,^3^ Ferreira et al,^9^ Karaçal et al,^13^ Hsu et al,^14^ Bhatnager et al,^5^ Chen et al,^8^ Tan et al,^7^ Coskunfirat et al,^12^ Lee et al,^15^ Spyropoulou et al^16^	118
Tissue adhesive + SG/flap	Morris et al,^20^ Sivrioğlu et al^21^	22

*SG indicates skin graft.
